# Health Information System and Health Care Applications Performance in the Healthcare Arena: A Bibliometric Analysis

**DOI:** 10.3390/healthcare10112273

**Published:** 2022-11-12

**Authors:** Ayogeboh Epizitone, Smangele Pretty Moyane, Israel Edem Agbehadji

**Affiliations:** 1 ICT and Society Research Group, Durban University of Technology, Durban 4001, South Africa; 2 Department of Information and Corporate Management, Durban University of Technology, Durban 4001, South Africa; 3 Honorary Research Associate, Faculty of Accounting and Informatics, Durban University of Technology, Durban 4001, South Africa

**Keywords:** health information system, health care applications, healthcare, HIS applications

## Abstract

There have been several studies centred on health information systems with many insights provided to enhance health care applications globally. These studies have provided theoretical schemes for fortifying the enactment and utilisation of the Health Information System (HIS). In addition, these research studies contribute greatly to the development of HIS in alignment with major stakeholders such as health practitioners and recipients of health care. Conversely, there has been trepidation about HIS’ sustainability and resilience for healthcare applications in the era of digitalization and globalization. Hence, this paper investigates research on HIS with a primary focus on health care applications to ascertain its sustainability and resilience amidst the transformation of the global healthcare space. Therefore, using a bibliometric approach, this paper measures the performance of health information systems and healthcare for health care applications using bibliometric data from the web of science database. The findings reveal solid evidence of the constructive transformation of health information systems and health care applications in the healthcare arena, providing ample evidence of the adaptation of HIS and health care applications within the healthcare arena to the fourth industrial revolution and, additionally, revealing the resilient alignment of health care applications and health information systems.

## 1. Introduction

The health information system (HIS) has gained enormous admiration within the healthcare arena and the global space over the decades [1,2]. This can be attributed to the advancement of technological deployment, global health priorities and universal health coverage. Remarkably, HIS and health care applications concepts’ representation in health research is not considered to be indispensable in the enhancement of health care [2,3], and it is emulated as a contrivance for health information assimilation and dissimilation efficacy and the integration of different units. Extant literature postulates information on patient needs and health care services core constituents of HIS despite the different definition of HIS instituted in different countries and organizations [4]. According to Jabareen and Khader [5], HIS is explained as an information design and build that aids health care applications, specifically, the management and utilization of health information. Similarly, extant literature posits HIS to be an expedient system that processes health data to provide information and knowledge within the healthcare arena [6,7].

Much of the adoption of HIS has been instated for several reasons, among which is included the enhancement of healthcare applications [5,8,9,10]. Data coverage that enabled health care programs takes the centre stage in health research, and it is heralded to be a critical component that structures HIS and supports timely decision making [11]. The position of HIS is not only vital for the enhancement of health care applications but also relevant to the attainment of global initiatives. Bhattacharya and Umar [12] highlight the contributions of HIS in monitoring progress and facilitating course correction for the Sustainable Development Goals (SGD) and countdown to 2030.

Although there has been ample evidence of health care studies in so many contexts, it is interesting to note that this does not measure health care performance. Extant literature reveals that, contrary to the enormous evidence of health care applications in the healthcare arena, there are major gaps in the delivery of quality health care [13]. A study that focuses on the countdown to 2030 and universal coverage precisely identifies essential health care inputs such as supplies, diagnostics and care content, as in healthcare provisions as determinants of health care applications [13]. However, despite this premise, practical evidence posits health care to be isolated within the healthcare arena [14]. Many authors aver the isolation of health care and indicated that it is neglected in healthcare delivery systems [14,15,16]. In order to afford quality healthcare delivery, integration is essential. The integration of different information systems is heralded to be indispensable, with the development of a resilient and sustainable HIS for an enhanced health care application.

Ammenwerth and Duftschmid [1] reveal the use of HIS’s instances, such eHealth, exclusively to support patient care and highlight the role they play in unifying silo systems to afford streamlined health care information exchange in the healthcare arena. However, prevailing literature reveals that the optimization of HIS adoption and use is reliant on the involvement of stakeholders’ perspectives, assessments and decisions [17]. Furthermore, extant literature highlights the absence of active engagement in HIS design despite its successful intervention in health care application [18]. Meanwhile, Zhang and Chen [19] highlight multisector collaboration and strengthening HIS as an urgent need within the healthcare arena.

Many health care applications in the healthcare arena have experienced added value from the use of HIS, among which includes improved population health, accurate resource distribution and management capacity [2,19]. Nevertheless, studies revealed that there are still questions and concerns about the potential use and alignment of HIS with certain health care applications’ objectives [20,21,22,23]. Ncube and Mars [24] argue that the lack of focus is a barrier that hinders HIS’s acknowledgement, alluding that most initiatives are fragmented and stalled. Sligo and Gauld [25] posit HIS’s enactment for health care applications to be inundated with glitches that have resulted in rife implementation flaws and failures. In addition, current literature conveyed that despite technological deployment being instituted to ameliorate problems within the healthcare arena, there is still insufficient attention devoted to the deployment of such technologies [3,21,24,26].

A study by Clay-Williams and Braithwaite [27] on resilient health care identified, among other determinants, the failure of HIS in the enhancement of health care applications, associating the lack of core stakeholders’ consideration on the safety of the system. Similarly, Samra and Li [28] reported HIS’ design and implementation to be flawed and identified determinants such as ethical, financial and functionality as barriers to its adoption and utilization within the healthcare arena. These authors allude to the shortcoming of HIS’s performance in health care within the healthcare arena [27,28]. Additionally, Kpobi and Swartz [29] stated that the apparent benefits of HIS enactment may be compromised by various determinants such as inadequate resources, thus substantiating the need to ascertain the performance of HIS and health care applications within the healthcare arena.

Healthcare is posited in extant literature to be a complex adaptive system with more than one intermingling and interdependent component, such as humans, technology and equipment [25,27,30,31]. Scholars maintained that this complex and dynamic nature influences outcomes within the healthcare arena [27,32]. Thus, there is a need to consider different variations in health care applications in the enactment of a sustainable and resilient HIS [27,33]. These authors collectively contended that incorporating and acknowledging the proposition of the healthcare system as dynamic with sociological components such as emergent, partially predictable and unknowable causality is unavoidable for effective health care enactment within the healthcare arena. This consensus is evident in the extant literature, as scholars posit that the interventions within the healthcare arena to ameliorate local problems are influenced unpredictably [27,34], indicating that anticipating and tackling these phenomena in the healthcare arena for optimal attainment of health care is necessary [25,35].

Regardless of the disposition of HIS and health care, Rudd and Puttkammer [30] assert HIS to be a crucial element of the global health systems’ development and fortification that necessitates innovative approaches to its utilization. Moreover, scholars revealed the need for a thoughtful and evidence-informed strategy to develop the right solutions in the healthcare arena [5,24,32], thus indicating the need for studies that anchor on the extant body of gen on HIS and health care applications to be explored. Hence, this study seeks to explore the performance of HIS and health care applications within the healthcare arena to append to the existing body of knowledge. Considering that the performance knowledge of the HIS and health care applications in the healthcare arena can contribute significantly to the enactment of a robust and sustainable HIS amid the uncertain globalization and digitalization, this study sought to ascertain the performance of HIS and healthcare applications in healthcare studies and to uncover the transformation in alignment with digitalization in the fourth industrial revolution.

## 2. Materials and Methods

### 2.1. Search Strategy and Inclusion Criteria

In this study, a bibliometric methodology that encapsulates the application of quantitative techniques was adopted [36,37]. This methodology is asserted to afford a concrete footing for the encroachment of an area in an innovative and epigrammatic way to facilitate and endow scholars to attain a holistic overview, new ideas for exploration, pinpoint knowledge gaps and position envisioned contributions in a field [36]. There are two bibliometric analysis techniques, namely: performance analysis and scientific mapping [36]. The performance analysis technique is used to account for the influences of the research components, such as publications, authors, journals, countries and institutions, in this study [36,38]. This analysis is asserted to be the hallmark of bibliometrics and evocative in nature [36]. It has been used in extant literature to analytically present empirical research in accordance with standard review practices [8,26,39,40,41,42]. The bibliometric method is apt for this study due to its ability to decrypt and map amassed knowledge and tinges of the established field to afford insight from an enormous volume of unstructured data in a robust mode, hence, the reason for its adoption [36]. Correspondingly, it aligns with similar studies in the healthcare and health information system arena that have afforded great findings [26,39,43,44,45].

The materials used in this study consist of study publications from 2013 to 2022, amassed using a total sampling technique for the subsequent bibliometric analysis. In the dataset key, bibliometric variables such as the publication title, author, abstract, keywords, publication year, publisher journal, type of publication and affiliations are among the variable examined. The search strings: “Health Information Systems” OR “HIS” AND “Healthcare” OR “Health care” AND “Application” OR “Applications” were used on the Web of Science database to extract data needed for the study, with attention given to their meaning within the study context. The web of science database was selected for this study due to its rich composition of bibliometric data over other databases such as Scopus, PubMed and MEDLINE. The query was refined several times to include the health care and HIS related areas and contexts. Only articles, reviews and conference proceeding papers were included, and the language was limited to English.

The final query search and link included “Health Information Systems” OR “HIS” AND “Healthcare” OR “Health care” AND “Application” OR “Applications” (All Fields) and Health Care Sciences Services or Public Environmental Occupational Health or Medical Informatics or Information Science Library Science or Infectious Diseases or General Internal Medicine or Research Experimental Medicine or Radiology Nuclear Medicine Medical Imaging or Integrative Complementary Medicine or Medical Laboratory Technology or Dentistry Oral Surgery Medicine or Biomedical Social Sciences or Emergency Medicine or Tropical Medicine (Research Areas) and 2022 or 2021 or 2020 or 2019 or 2018 or 2017 or 2016 or 2015 or 2014 or 2013 (Publication Years) and English (Languages) and Article or Review Article or Proceeding Paper (Document Types) and Public Environmental Occupational Health or Health Care Sciences Services or Medical Informatics or Health Policy Services (Web of Science Categories) and Article or Review Article or Proceeding Paper (Document Types) and Health Care Sciences Services (Web of Science Categories) and Health Care Sciences Services or Public Environmental Occupational Health (Web of Science Categories).

### 2.2. Study Selection

The bibliometric analysis included data that fit within the study context and adhere to the delineation of HIS, healthcare and health care applications. For this study, HIS is considered to be a unified system that constitutes one or more interrelated components such as technology, people and processes that aid health care delivery. Meanwhile, healthcare and health care, though often used interchangeably, referred to the enhancement of health through engagements such as prevention, diagnosis, treatment and cure for illness and or diseases from health care providers [27], and healthcare/health care application refers to the mode employed for the delivery of health services within the healthcare arena. Thus, publications that covered any component and dimension of the search strings with at least one cog related to HIS instances for health care were selected in alignment with the context delineation regardless of the ambiguity and vague applications of these terms within healthcare, and only articles, review articles and conference proceedings that were written in English were selected. The initial screening was based on the title and abstract and was limited from 2013 to 2022. Exclusion criteria that were exercised included the elimination of publications that were not written in English and those that were retracted.

**Figure 1:**
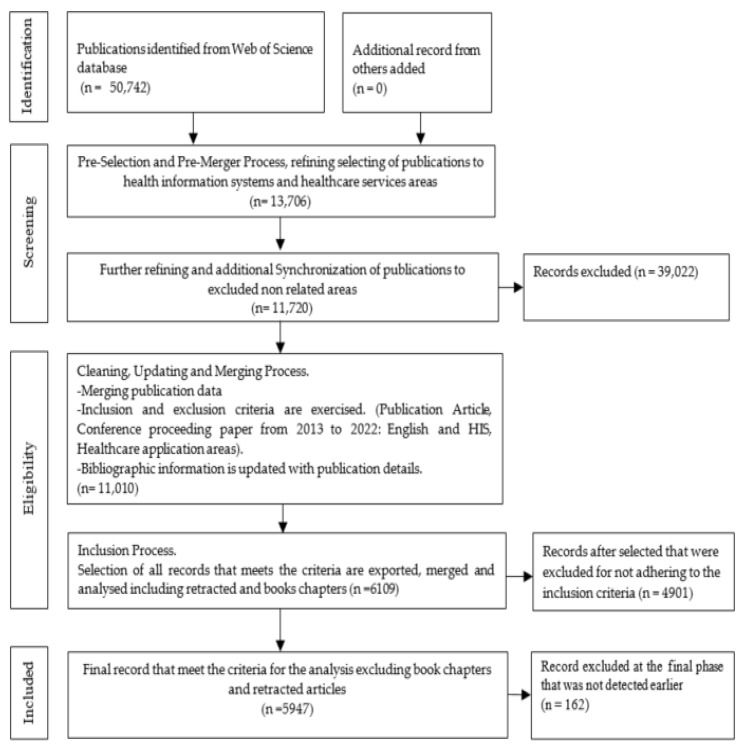
Study methodology.

Figure 1 illustrated the different phases conducted to consolidate the data for the bibliometric analysis. The downloaded data were pre-processed and consolidated into one file and then imported to the R studio version 2022.07.1 Build 554. The bibliometrix package and biblioshiny functions were used for the bibliometrix analysis. More details about our methodology are available via the GitHub link: https://github.com/ayo-prog/Biblio/blob/main/analysis/Rscript_pipeline.Rmd (accessed on 8 November 2022).

## 3. Results and Discussion

Aggregated data of 6109 articles were initially obtained from the Web of Science database, featuring articles on the health information system and healthcare themes. After a rigorous process, 5947 articles were retained that adhered to the inclusion and exclusion criteria. A bibliometric analysis was then conducted using the R programming language and its bibliometrix package to uncover, classify and identify the topic, trend and map performance. The mappings were then established via the incorporation of different exploratory components, namely, the keywords and units in the analysis, and were visualized to provide graphical illustrations of the density spots and themes.

The descriptive analysis revealed information from four main sections of the dataset, namely: the main information, document contents, authors’ collaboration and document types, and is shown in Table 1. It can be noted that the range from 2013 to 2022 has made significant contributions in the healthcare area. The growth in the HIS and health care applications arena revealed a 13.88% increase, which included 158 journal sources and 5947 documents with an average age of 3.58, also revealing the average citation per document to be 16.7 from a reference of 174,599.

**Figure 2:**
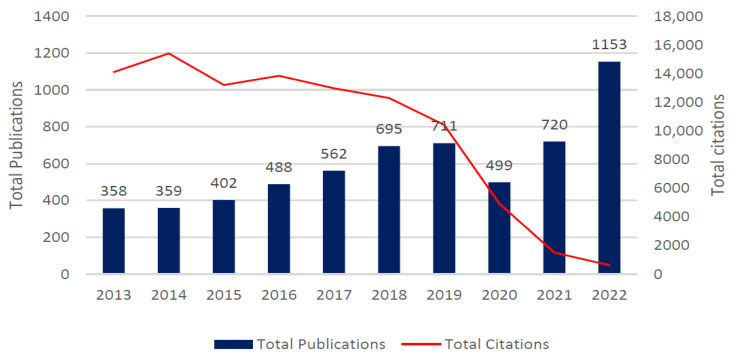
Total publications and total citations.

Figure 2 illustrates the distribution of the citations over the yearly publication, respectively. The document contents mainly constitute the keywords and show authors’ compositions to be 22,751 and 204 for authors of single-authored documents. The aggregated keywords consisted of the main keywords of 10,960 and extra plus keywords of 7575. The holistic representation of author collaboration indicated 234 counts associated with single authors’ documents and 5.49 for co-authors per document. In this category, the international co-authorship amounted to 27.32%. Lastly, the document types included 4887 articles, 976 reviews and 84 proceedings in paper articles.

**Figure 3:**
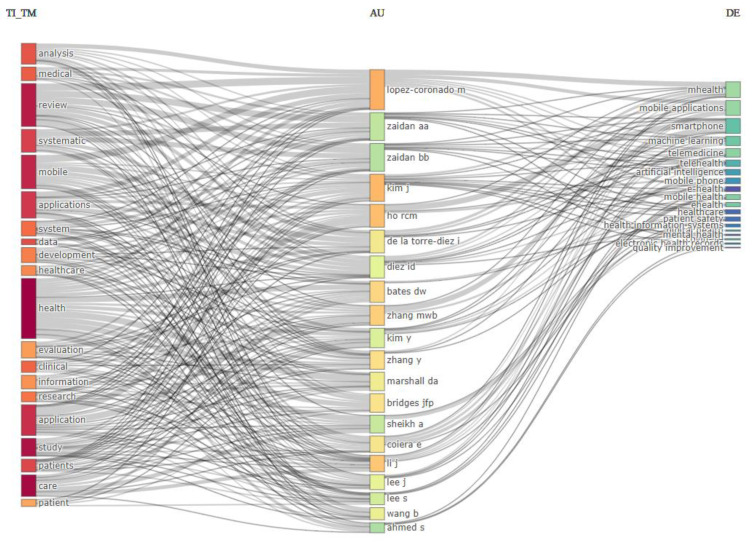
Three-field plot of title, authors and keywords.

The three-field plot of the titles, authors and keywords presents the correlation between the publications with the most prominent concepts of HIS and health care applications being captured. For the 20 included records, the HIS was revealed to have been only identified twice in contrast to the many related concepts and health care applications present (Figure 3).

Source analysis consisted of the most relevant and locally cited sources for the aggregated dataset. The most relevant and locally cited sources that reveal the documents’ title and source title that was cited, as per the references list for each document, measures the productivity in the health information and healthcare arena. For the most relevant sources, the journal of medical internet research was the most relevant source for health information systems and health care, with 476 documents, followed by the BMC health services research and JMIR m-health and u-health, with 317 and 310 documents in the area. These journals represent a greater scope and content for health information systems and healthcare studies. The greater focus and extent can still be seen in the most locally cited source in the area, with the consistent presence of the five most relevant sources featured in the most locally cited sources, three of which were the top most locally cited in the category. The journal of medical internet research has the most impact on the study as this journal clearly illustrated prodigious coverage in the health information and healthcare subject area. Similarly, these journals collectively performed prominently when associated with the h-index, g-index, m-index and total citations. However, some journals contributed fewer documents than others, for instance, the American medical informatics association had 207 documents with an impact greater than some having more documents, and was the fourth most impactful in the healthcare studies.

The citation per publication revealed a gradual rise in the number of citations per publication over the decade. The year 2022 is in a state of incompleteness and, as a result, its measurement does not count in this regard, while it can be ascertained that there will be high performance for the year 2022. The gradual rise over the years revealed the average performances of the respective journal publications for the decade to be truncated, despite the steady progression of publications per year. The years 2013 to 2019 show a gradual increase in the publications, with 2020 being the cut-off, and an upsurge from 2020 that has been maintained steadily to 2022, even with the citations plunging downward. The ten top sources in both categories can be seen in Table 2, which illustrates and details the different relevant journal sources and the documents in the source and how they aggregately contributed significantly to the knowledge of HIS and health care applications. The color peach was used to show the distribution of the 5 most relevant and locally cited sources. 

**Figure 4:**
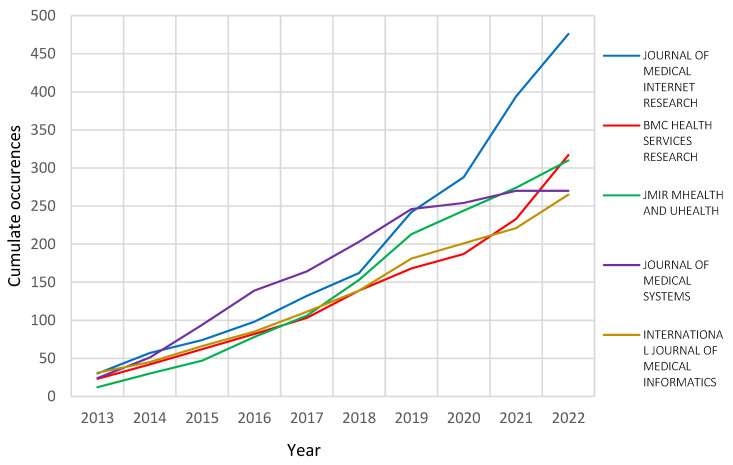
Source growth.

These aforementioned journals together indicate and demonstrate their impact contribution in the HIS and health care applications, thus positioning these sources to be the go-to sources in the area. Moreover, the source dynamics on the HIS in association with healthcare and health care applications also reveal the distribution of publication growth over the years from the different sources directly associated with the top impact sources. The source dynamics also show the growing interest and trends of publications across the identified sources based on cumulative numbers of publications on the HIS and health care applications in the healthcare arena studies, as shown in Figure 4.

In the authors’ analysis, the measurement of the production, performance and impact of the author’s contribution is determined by the relevance, citations, production over time, Lotka’s law and their impact, as indicated in Table 3. In the health information and healthcare applications arena, the most prominent author across all categories is classified by relevance, citations and impact. Across all categories, the most contributing author is Lopez-Coronado M. For the most relevant authors category, which quantifies the authors’ productivity and publications in the area, Lopez-Coronado M (22), followed by Kim J (18), HO RCM (16), Li L (16) and Zhang MWB (15), took a lead. However, this does not automate their impact, as it can be seen that most authors at the bottom of the most relevant category, such as Bridges JFP and De la Torre-Diez I, were among the top locally cited and influential authors based on the total citations. Even though Bridges JFP (1164) received the highest citation with 11 documents, the author was positioned below similar authors who had high indexes with the same document count.

**Figure 5:**
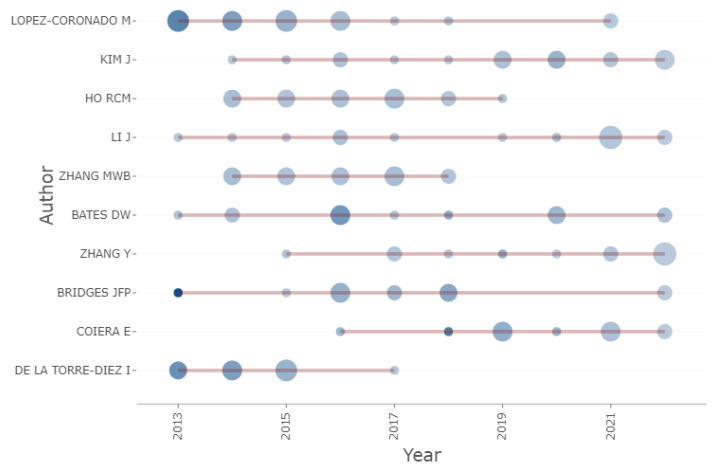
Top 10 authors’ production over time.

Similarly, these authors had the top production over time, as illustrated in Figure 5, and they produced more articles in the area. Figure 5 shows the author’s publication timeline in orange. Li J and Bridges JFP are among the authors with the longest timeline. The blue dots in the Figure 5 represent the number of documents published by these authors with one being the fewest and six being the most. The size and intensity of the dots on the timeline are proportional to the number of documents and total citations per year. Lopez-Coronado M created 5 documents in 2013 and received 47.5 citations. Bridges JFP had one document with a higher citation of 84.2 in the same year. Coiera E created a single document in 2018 with a citation on 52.2. In terms of Lotka’s Law, the authors’ productivity and relationship to their article were predicted to be 68.3% per document, thus indicating the most likely number of contributing authors to documents to be 15,532. The most cited local authors categories present authors who have been cited more by other authors in the dataset and healthcare area. The most cited local authors were Lopez-Coronado M (129), De La Torre-Diez I (111) and Martinez-Perez B (105). With regards to the authors’ impact category, across the time frame, Lopez-Coronado M was seen to be dominating in the HIS and health care applications arena.

**Figure 6:**
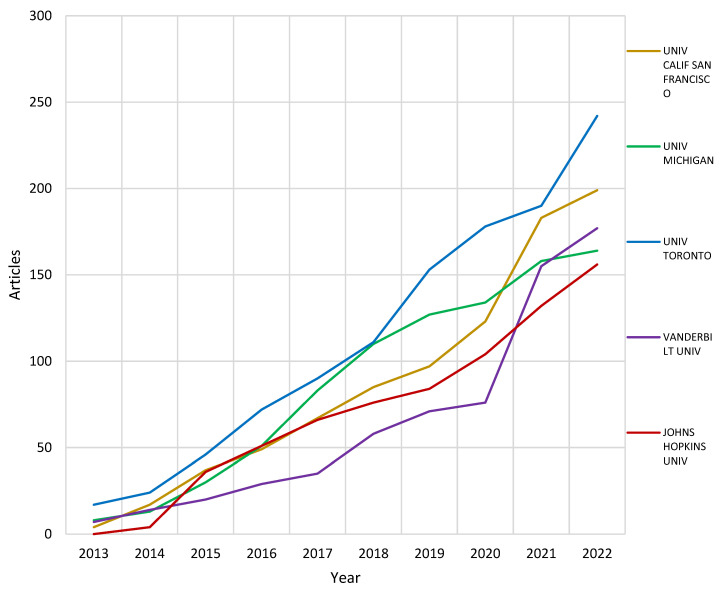
Affiliations’ production over time.

For the affiliations analysis, the most relevant, and production over time in this regard, is presented for the HIS for the health care applications milieu. The University of Toronto (242), California—San Francisco (199), Vanderbilt (177), Michigan (164) and Johns Hopkins (156) had the top affiliated authors, followed by other universities in the same region. Likewise, these universities dominated the top affiliations’ production from the 2013 to 2022 span, as presented in Figure 6.

**Figure 7:**
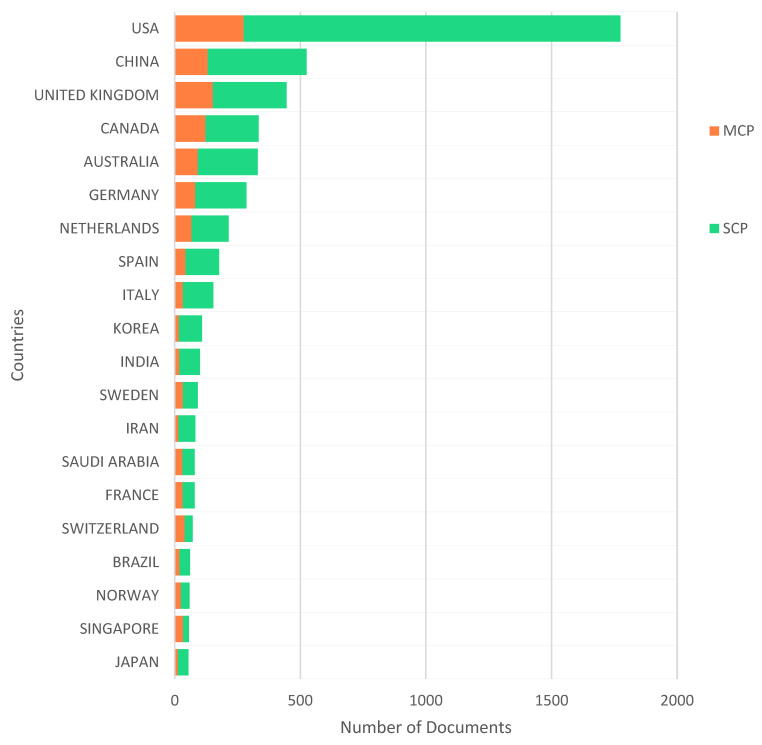
Countries’ MCP and SCP.

**Figure 8:**
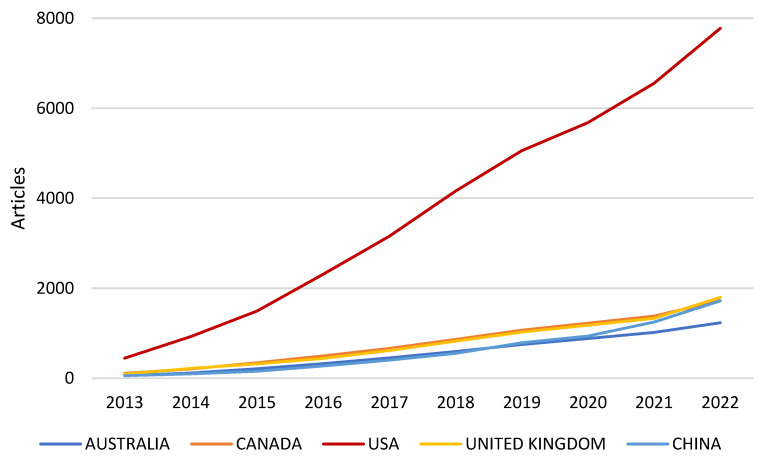
Countries’ production over time.

In the countries’ analysis of the corresponding author’s country, outputs for single-country publications (SCP) and multiple country publications (MCP) were dominated by developed countries with higher publications, namely the USA (1774), UK (446), Canada (334), Australia (331) and Germany (286), with China (525) being the only developing country in the top tier (see Figure 7). Some developing countries, such as India (101), Iran (80) and Saudi Arabia (80), were seen to be ascending gradually up the ladder in the HIS for health care applications. Likewise, these countries contributed greatly to the scientific production and general production in the HIS for the health care application milieu over the decades, as shown in Figure 8. Moreover, these countries, especially the USA (33,871), UK (10,680), China (7517), Australia (6775), Canada (6570) and Germany (3701), received the most citations within the arena, whilst, when looking at most cited countries by the average citations per year, not many of these mentioned countries received coverage in this regard. Countries such as Croatia (83), Uganda (320), Senegal (73), Qatar (365) and Zambia (176) were in the top tiers, posting 83, 80, 70, 40.5 and 35.2 publications.

**Figure 9:**
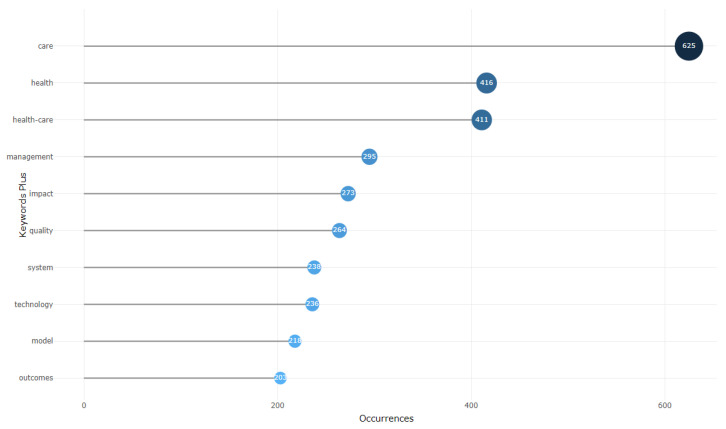
Most frequent words.

The documents analysis had three categories, namely: documents, cited references and words. The most globally cited document comprised the paper by Wan X, 2014 on an enhanced methodology for healthcare study, whereas the most locally cited document was by Deennison L, 2013. The aforesaid documents have become a major reference in the study of health information and healthcare application. In lieu of the word analysis, the frequent words in the HIS and health care applications were namely: care, health, health-care, management, impact, quality, system, technology, model and outcomes, as illustrated in Figure 9. These words occur more than 200 times in the health information and healthcare application studies, and similar words were also present in the titles and abstracts of many studies. A word cluster, which is a graphical illustration of word count in the area, shows these as the main themes that characterize the health arena. The “HIS”, “Healthcare”, “health care” and different instances such as m-health and telehealth were among many of the studies conducted.

**Figure 10:**
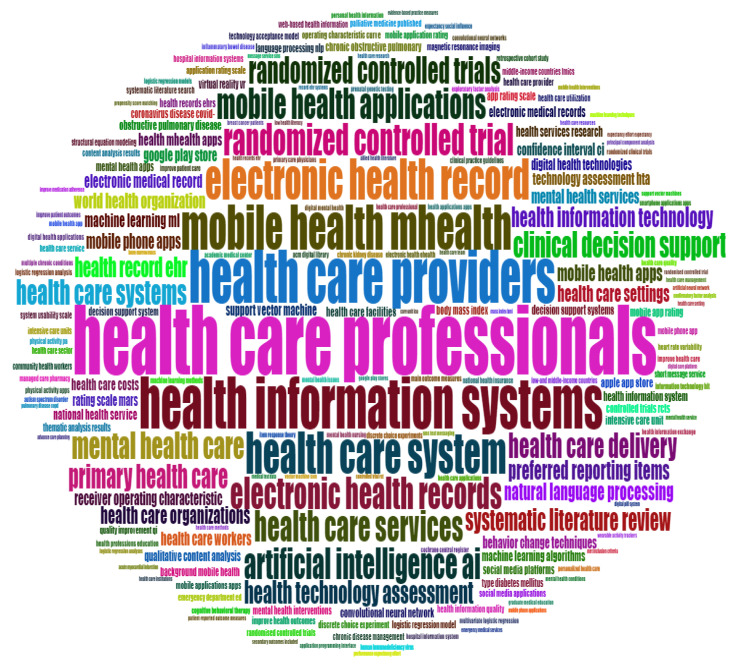
Word cloud based on the titles.

**Figure 11:**
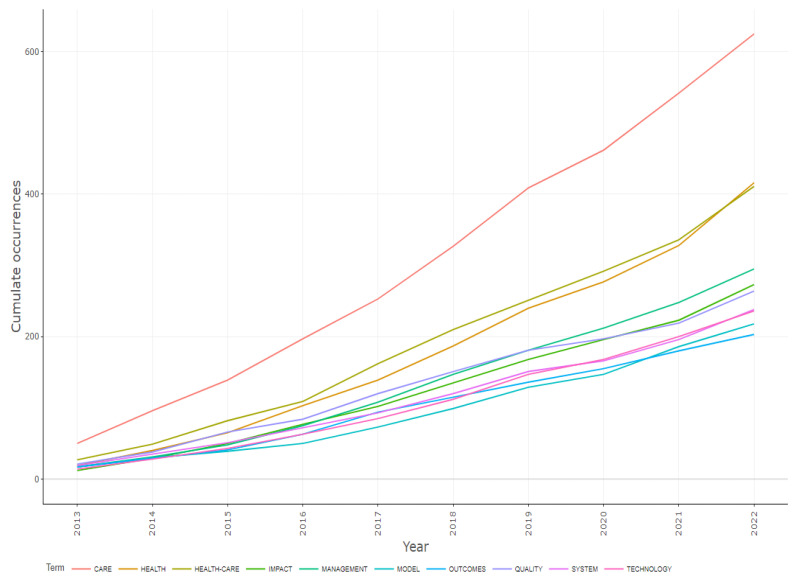
Word dynamics.

As indicated in Figure 9, over the years, these areas have attracted many researchers and continue to do the same, as they extend to many associated instances such as telehealth, m-health, e-health and health records. Although many of the same concepts have been gaining recognition over time, the current HIS and healthcare applications settings have extended these word themes, and more studies are emerging with a focus on tele-healthcare, digital health, mobile health applications and machine learning, artificial intelligence, clinical decision support and the health care system, services and providers in the health information area. Figure 10 shows the word cloud that illustrates the prominent themes in the area by presenting them as bigger than the other words in the area. Furthermore, the word dynamics show how the keywords in the area have evolved over the decade in the HIS and health care applications within the healthcare arena, as presented in Figure 11. This confirms a continuous evolvement in the health themes around care, healthcare and health that aligns with the fourth industrial revolution. Although areas such as the impact, management and quality in the health arena studies are also advancing, many foundational components such as technology, system, model and outcome are lagging.

**Figure 12:**
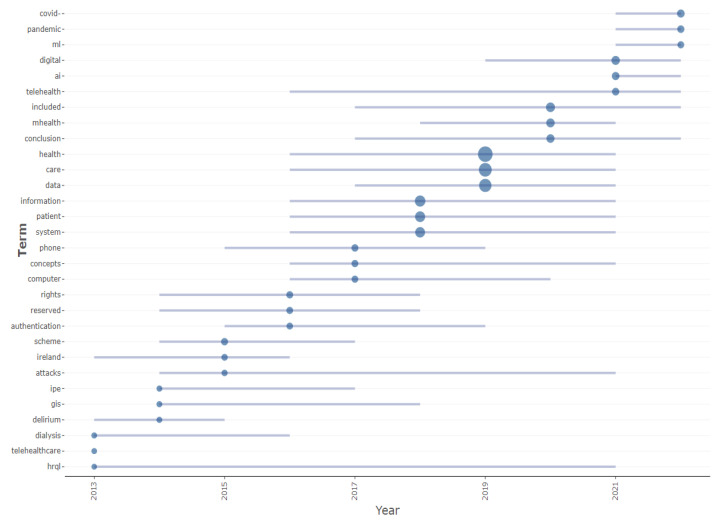
Trending topics.

Figure 11 shows the measurement of the growth base over the years for many healthcare arena concepts. These healthcare concepts have shaped health studies, revealing many trends in the arena. Though health care concerns such as anxiety, complications, family caregivers and generation have dominated healthcare arena studies, there is soon to be a technological revolution in healthcare arena studies. Key concepts such as machine learning, artificial intelligence and digitalization have gained momentum in health arena studies, contrasting studies that reveal a narrow focus on technology for health care [26]. The COVID pandemic, which is also trending in health arena studies, seems to have been the catalyst for many of these trends, notwithstanding, over the decades, the HIS and health care applications have remained relevant and pertinent in the health arena, as depicted in the trend graph shown in Figure 12. Figure 12 depicts the evolution of the trending concepts using a blue line that indicate the commencing year to the final year. Correspondingly using the blue dots represent the total frequency of these concepts in the median year.

To provide health care interventions with the attention they deserve, a sustainable and resilient HIS is required as well as a well-defined health priority globally [18,46]. The transformation and performance of HIS and health care applications in the healthcare arena are considered to be favourable over the decades, despite the uncertainty within the global space [47]. Initiatives to inform the enhancement of the quality of care afforded by healthcare providers are progressively flouted in order to sustain a high quality of care amidst amassed demand and financial shortcuts [48]. However, the results indicate multiple measures over the years employed to address these challenges. This aligned with prior studies that highlight the need for best practices and improvement in global health settings [46,49]. The trends, keywords and content of the HIS and health care applications in the healthcare studies illustrate the high performance of this content in attending to hurdles in the health arena [50,51,52,53,54,55], thus revealing a refined HIS that, over time, has emerged to be resilient and sustainable, with many instances for health care applications such as eHealth, mHealth, telemedicine and telehealth. The findings highlight the performance of HIS and health care applications to comprehend the global perspective on health. Additionally, this constitutes one important step toward going beyond solely unanalytical upshots in refining the quality of care today. This is in line with previous studies that reveal that HIS has the potential to stimulate the move beyond traditional HIS enactment for health care applications within the healthcare arena. Although prior studies within the healthcare arena context highlight a constricted technological emphasis in health care [26], the findings of this study revealed the contrary, illustrating a variation in technological applications in the healthcare arena. However, El Khatib and Hamidi [56] report the pace of healthcare in embracing digital transformation and disruption to be slow, implying that the need for healthcare stakeholders to leverage digitalization within the healthcare arena is paramount.

## 4. Conclusions

The healthcare arena is a dynamic environment that constantly deals with many ambiguous actors, which influences many of its constituents. HIS and health care applications are expected to embrace this setting to present help for many stakeholders. The body of gen in HIS and health care applications provides ample evidence that the performance of these constituents in the healthcare arena is greatly significant. Hence, this study was particularly attentive to the sustainability and resilience of HIS for health care applications over the last decade, and the study findings demonstrate the retrospective concepts in the healthcare arena, concomitant with HIS and health care applications globally. Many concepts such as telehealth and m-health have emerged in direct response to health concerns such as COVID, indicating the enrichment of the HIS and health care applications in extant literature. The collegial and pragmatic accomplishments within the HIS and health care applications which illustrate a meaningful impact in the healthcare arena were apparent, as well, wherein scholars’ contributions were ascertained and advances were made that indicate the capabilities of HIS for health care applications in the healthcare arena. The findings further unearth the sustainable and resilient capabilities embedded within HIS and health care applications in the healthcare arena. Additionally, this finding can inform stakeholders such as decision-makers, policy-makers and health organizations on future directions and developments of HIS for health care applications that incorporate sustainable developmental goal 3, associated with the enactment of good health and wellbeing for all. Future research that delves more within the context to uncover the intellectual and conceptual structure of HIS for health care application in the healthcare arena is needed. Furthermore, more databases can be included to provide a detailed synthesized result that can contribute to the attainment of quality health care in different settings.

## Figures and Tables

**Table 1:** Main information.

Description	Results
Main Information about Date	
Timespan	2013:2022
Sources (Journals)	158
Documents	5947
Annual Growth Rate %	13.88
Document Average Age	3.58
Average citations per doc	16.7
References	174,599
Document Contents	
Keywords Plus (ID)	7575
Author’s Keywords (DE)	10,960
Authors	
Authors	22,751
Authors of single-authored docs	204
Authors Collaboration	
Single-authored docs	234
Co-Authors per Doc	5.49
International co-authorships %	27.32
Document Types	
Article	4887
Article; proceedings paper	84
Review	976

**Table 2:** The most relevant and local cited sources.

Most Relevant Sources		Most Local Cited Sources		Source Impact			
Sources	Articles	Sources	Articles	Sources	h- index	g- index	m-index
JOURNAL OF MEDICAL INTERNET RESEARCH	476	J MED INTERNET RES RESEARCH	5293	J MED INTERNET RES	60	106	6
BMC HEALTH SERVICES RESEARCH	317	J AM MED INFORM ASSN	2610	JMIR MHEALTH AND UHEALTH	47	70	4.7
JMIR MHEALTH AND UHEALTH	310	JMIR MHEALTH UHEALTH	2557	J MED SYST	43	61	4.3
JOURNAL OF MEDICAL SYSTEMS	270	PLOS ONE	2471	J AM MED INFORM ASSN	41	69	4.1
INTERNATIONAL JOURNAL OF MEDICAL INFORMATICS	265	JAMA-J AM MED ASSOC	2279	INT J MED INFORM	39	61	3.9
JOURNAL OF HEALTHCARE ENGINEERING	246	INT J MED INFORM	2149	BMC HEALTH SERVICES RESEARCH	29	50	2.9
TECHNOLOGY AND HEALTH CARE	221	NEW ENGL J MED	1868	TELEMEDICINE AND E-HEALTH	29	46	2.9
JOURNAL OF THE AMERICAN MEDICAL INFORMATICS ASSOCIATION	207	J MED SYST	1675	ACADEMIC MEDICINE	25	40	2.5
HEALTHCARE	162	BMJ-BRIT MED J	1565	HEALTH AFFAIRS	25	46	2.5
TELEMEDICINE AND E-HEALTH	160	LANCET	1533	IMPLEMENTATION SCIENCE	25	56	2.5

**Table 3:** Authors analysis.

Authors Analysis									
Most Relevant		Most Local Cited		Impact					
Authors	Articles	Author	Local Citations	Element	h-Index	g-Index	m-Index	TC	NP
LOPEZ-CORONADO M	22	LOPEZ-CORONADO M	129	LOPEZ-CORONADO M	12	22	1.2	942	22
KIM J	18	DE LA TORRE-DIEZ I	111	ZAIDAN AA	11	11	1.222	580	11
HO RCM	16	MARTINEZ-PEREZ B	105	ZAIDAN BB	11	11	1.222	580	11
LI J	16	BRIDGES JFP	65	BATES DW	10	14	1	483	14
ZHANG MWB	15	CHRISTENSEN H	62	BRIDGES JFP	10	11	1	1164	11
BATES DW	14	YARDLEY L	55	DE LA TORRE-DIEZ I	10	13	1	787	13
ZHANG Y	14	MANDL KD	54	HO RCM	9	15	1	240	16
BRIDGES JFP	13	BENDER JL	53	MARSHALL DA	9	11	1.125	397	11
COIERA E	13	PROUDFOOT J	52	MARTINEZ-PEREZ B	9	10	0.9	723	10
DE LA TORRE-DIEZ I	13	JOHNSON FR	48	ALBAHRI AS	8	8	1.6	475	8
